# Steam explosion enhances phenolic profiles and antioxidant activity in mung beans

**DOI:** 10.1002/fsn3.2711

**Published:** 2022-02-22

**Authors:** Fachun Wan, Chunyu Hou, Kaiyun Luo, Anwei Cheng

**Affiliations:** ^1^ 12575 College of Animal Science and Technology Hunan Agricultural University Changsha China; ^2^ 74641 Institute of Agro‐food Science and Technology Shandong Academy of Agricultural Sciences Jinan China; ^3^ 12575 College of Food Science and Technology/Engineering Center of Rapeseed Oil Nutrition Health and In‐depth Development in Hunan Province Hunan Agricultural University Changsha China

**Keywords:** antioxidant activity, mung bean, phenolics, steam explosion

## Abstract

Steam explosion (SE), as a physicochemical pretreatment process, has the dual effect of high temperature and high pressure. In this study, SE was applied to pretreat mung beans to increase phenolic extraction and their antioxidant activity. It can make the material loose and porous, which is beneficial to the release of phenolic compounds from mung beans. Insoluble‐bound phenolics (IBPs) were the dominating fraction, followed by glycosidic phenolics (GPs) and esterified phenolics (EPs), and free phenolics (FPs) were the lowest in mung beans. After SE, the maximum contents of FPs, EPs, GPs, IBPs, and total phenolics were detected at 0.75 MPa for 30 s, which were 1.47‐, 1.87‐, 1.73‐, 1.48‐, and 1.58‐fold compared with the untreated samples, respectively. On the whole, the effect of SE on phenolics in mung beans first increased and then decreased. SE increased the contents of protocatechuic acid, *p*‐coumaric acid, ferulic acid, catechin, and epicatechin; but there was a decrease in caffeic acid. Compared with the untreated samples, the antioxidant activity of FPs, GPAs, EPs, and IBPs was also improved by SE. The relationship between the phenolic content and antioxidant activity was very high with coefficients of 2,2′‐azinobis (3‐ethylbenzothiazoline‐6‐ sulfonic acid) > 2,2′‐diphenyl‐1‐picrylhydrazyl > ferric reducing antioxidant power. In conclusion, an appropriate SE can lead to a more efficient extraction of phenolics and improvement of antioxidant activity in mung beans.

## Graphical abstract

Steam explosion (SE) formed the large cavities and intercellular spaces of mung beans. SE increased the yields of insoluble‐bound (IBPAs), glycosided (GPAs), esterified (EPAs) and free phenolic acids (FPAs). SE enhanced the antioxidant activity of IBPAs, GPAs, EPAs, FPAs in mung beans.

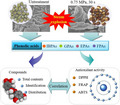

## 1 INTRODUTION

Legumes, a group of crops that produce pods, are important resources of food and feed. Up to now, there are no more than 20 legume crops widely cultivated in the world. Among them, mung bean (*Vigna radiata* L.) is a commercial legume crop widely planted in tropical and subtropical areas, such as China, India, Korea, Japan, and Thailand. Mung bean can be made into bean paste, cake, and vermicelli (Anwar et al., 2007). In addition, it has medicinal effects, such as detoxification, diuresis, and eyesight. So, mung bean is currently considered a rich source of both nutrients and bioactive compounds. Phenolics are a class of bioactive compounds found in plants and have some potential activities to promote human health. Mung beans are rich in phenolics which were mainly studied to contain *p*‐coumaric acid, ferulic acid, gallic acid, catechin, quercetin, caffeic acid, kaempferol, chlorogenic acid, syringic acid, neochlorogenic acid, and so on (Pajak et al., 2014; Shi et al., 2016; Hung et al., 2020; Yao et al., 2013; Zhang et al., 2013). Reports have demonstrated that mung bean extracts have some functional activities, such as anti‐inflammatory (Lee et al., 2011), antidiabetic (Lee et al., 2011; Peng et al., 2008), antiproliferative (Yang et al., 2020), and antioxidant activities (Shi et al., 2016; Wang et al., 2016). Furthermore, it has been confirmed that phenolics rich in mung beans are closely related to the above activities (Shi et al., 2016; Wang et al., 2016).

Phenolics generally occur as the forms of free/soluble conjugated and insoluble/bound in plants (Shi et al., 2016), but mainly are in an insoluble/bound form in nature (Shi et al., 2016; Wang et al., 2011). Insoluble phenolics are covalently bound to cell wall structural components through ester, ether, or acetal bonds (Wang et al., 2011; Xu et al., 2008). Many in vitro antioxidant assays have shown that both free and bound phenolics have a significant antioxidant activity. But some reports have confirmed that antioxidant activity of insoluble/bound phenolics was stronger compared with the free form (Chandrasekara & Shahidi, 2010; Liyana‐Pathirana & Shahidi, 2006; Wang et al., 2011).

Steam explosion (SE), classified as a physicochemical pretreatment process, has several characteristics, such as acid‐based hydrolysis, thermal degradation, mechanical fracture, hydrogen‐bond destruction, and structural rearrangement (Li et al., 2020). Explosion temperature/pressure and residence time are considered the main factors of SE used in the extraction of bioactive compounds. The optimal explosion condition for the extraction of free and bound phenolics and the improvement of their antioxidant activity in adzuki beans was at the pressure of 0.75 MPa for 90 s (Cheng et al., 2020). The maximum content of free phenolics, total soluble conjugate phenolics, and their antioxidant activity in barley bran was detected under the SE of 250°C for 120 s (Gong et al., 2012). Phenolic compounds and their antioxidant activity in soybean seed coats were obviously increased by using steam flash explosion (Chen et al., 2020). SE has the synergy of high temperature and high pressure, and the effect on polyphenols is more obvious than that of a single temperature or pressure.

The compositions and biological activity of phenols in mung beans have been reported (Shi et al., 2016; Wang et al., 2016; Yao et al., 2013; Zhang et al., 2013), but there is no comprehensive study on the extraction of phenolics with free, esterified, glycosidic, and insoluble‐bound forms in mung beans after SE. Accordingly, it is necessary to determine their antioxidant activity by 2,2′‐diphenyl‐1‐picrylhydrazyl (DPPH), 2,2′‐azinobis (3‐ethylbenzothiazoline‐6‐ sulfonic acid) (ABTS), and ferric reducing antioxidant power (FRAP) assays. So, in this paper, SE experiments were carried out, at different pressures with different residence times, to investigate the compounds of phenolics and their antioxidant activity in mung beans.

## 2 MATERIALS AND METHODS

### 2.1 Chemicals

All standards including gallic acid, protocatechuic acid, catechin, caffeic acid, epicatechin, *p*‐coumaric acid, ferulic acid were purchased from Sigma‐Aldrich. Reagents of Folin–Ciocalteu phenol and DPPH were from Shanghai Yuanye Biology Co., Ltd. The assay kits of ABTS and FRAP were from Beyotime Biochemistry Co., Ltd. Other chemical reagents are of analytical grade.

### 2.2 Materials

Harvested mung beans (*V. radiata* L.; Variety: No. 8 Bailv) were obtained from the local Zhangqiu Farm, Shandong province, China. Beans were naturally dried in the Sun to <8% H_2_O.

### 2.3 Steam explosion pretreatment

The SE of mung beans was performed using a QBS‐300 batch SE apparatus (Hebi Gentle Bioenergy Ltd) (Cheng et al., 2020). The explosion pressure in reactor was carried out at 0.25, 0.50, 0.75, and 1.0 MPa (the corresponding temperature being 127.4, 151.0, 167.5, and 179.0°C) for 30 and 90 s, respectively. The explosion samples were dried at 50°C to reach a constant weight and then crushed to 60 mesh. The sample without SE treatment was the control.

### 2.4 Structural observation

Powdery beans with different SE conditions were sputter‐coated with gold. At an accelerating voltage of 5.0 kV with the vacuum condition, microstructure of samples was observed by scanning electron microscopy (JSM‐6700F, JEOL).

### 2.5 Preparation of crude extracts from mung beans

Powdery beans were mixed with 50% ethanol at a solid–liquid ratio of 1:40 (g:ml), sonicated at 300 W for 1 h, and then followed by centrifugation at 1300 *g* for 15 min. The extraction was carried out twice and combined the supernatants, which would be used for the separation of free phenolics (FPs), esterified phenolics (EPs), and glycosidic phenolics (GPs); their residues were reserved for the extraction of insoluble‐bound phenolics (IBPs).

### 2.6 Separation of differently bound phenolics from mung beans

**FIGURE 1:**
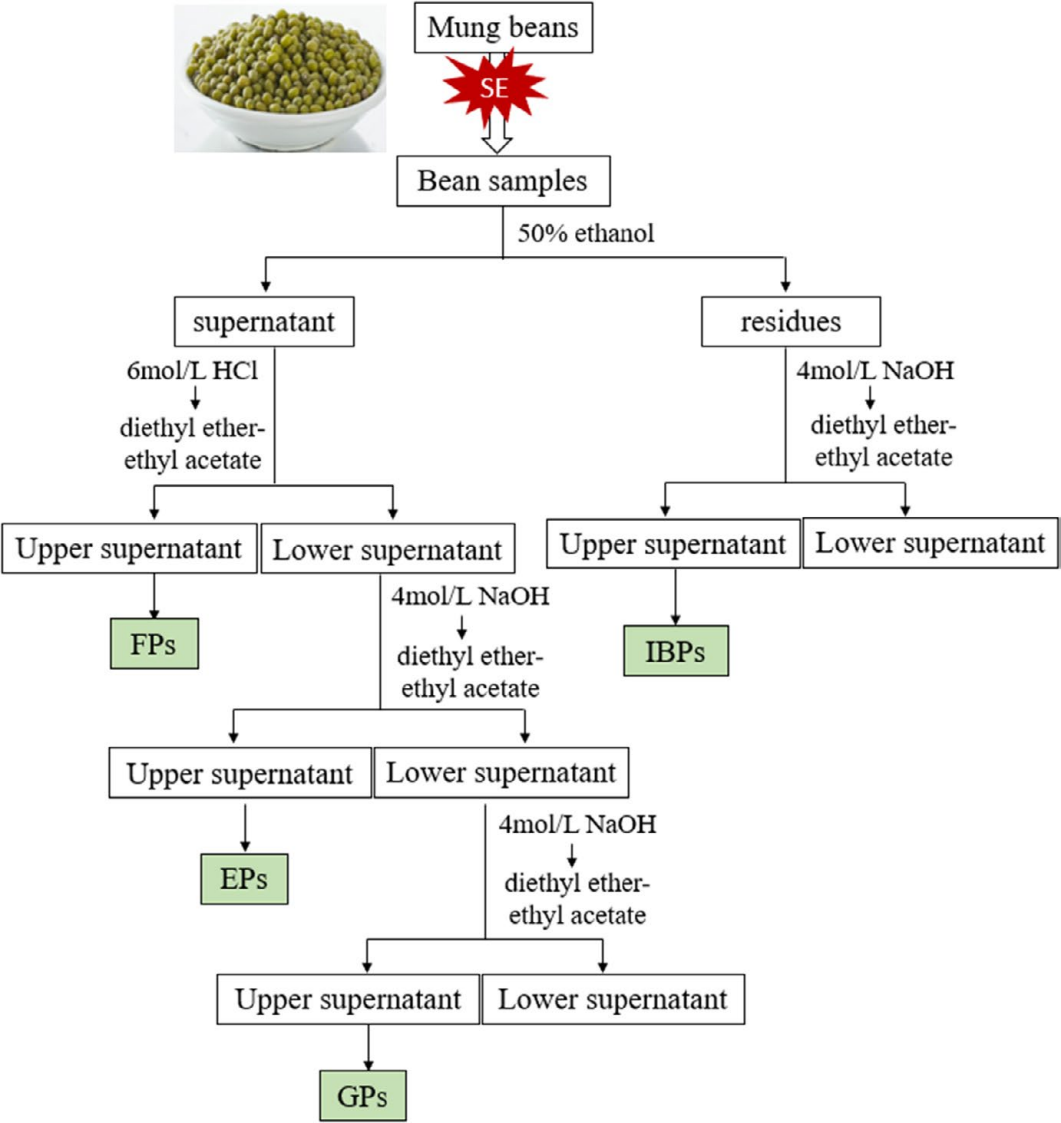
Schematic diagram of phenolics extraction

The separation of FPs, EPs, and GPs from crude extracts was performed according to the methods of Wang et al. (2011), and their extraction process is shown in Figure 1.

(1) Preparation of FPs: The crude extracts were adjusted to pH 2 by using 6 mol/L HCl, followed by mixing with the equal volume of diethyl ether‐ethyl acetate (1:1, v/v). The extraction was performed six times and the diethyl ether‐ethyl acetate extracts are combined to obtain FPs.
(2) Preparation of EPs: The aqueous phase was hydrolyzed with 4 mol/L NaOH (containing 10 mmol/L ethylene diamine tetraacetic acid [EDTA] and 1% ascorbic acid) under N_2_ for 4 h at room temperature, and then acidified to pH 2 with 6 mol/L HCl. The hydrolyzed solution was mixed with an equal volume of diethyl ether‐ethyl acetate (1:1, v/v). The process was performed six times and combined with the diethyl ether‐ethyl acetate extracts. EPs were obtained from the diethyl ether‐ethyl acetate extracts.
(3) Preparation of GPs: The remaining aqueous phase in the above was hydrolyzed with 5 ml of 6 mol/L HCl at 85°C under N_2_ for 30 min. The hydrolyzed solution was mixed with diethyl ether‐ethyl acetate (1:1, v/v) at a ratio of 1:1, and performed six times. The diethyl ether‐ethyl acetate extracts are combined to obtain GPs.
(4) Preparation of IBPs: IBPs from mung beans were prepared by the alkaline hydrolysis method (Cheng et al., 2014). At room temperature, the residues from 50% methanol extractions were mixed with 4 mol/L NaOH at solid–liquid ratio (g:ml) of 1:20 and hydrolyzed for 4 h under a stream of N_2_. The mixture was adjusted to pH 2 with 6 mol/L HCl and centrifuged at 3000 *g* for 15 min. The hydrolyzed solution was mixed with an equal volume of diethyl ether‐ethyl acetate (1:1, v/v). The process was performed six times, and the diethyl ether‐ethyl acetate extracts are combined to obtain IBPs.

The above extracts of four fractions were evaporated at 45°C and subsequently dissolved in 80% methanol. The phenolic content was determined by Folin–Ciocalteu colorimetric method and phenolic compounds were identified by HPLC.

### 2.7 Determination of phenolic content

Phenolic contents in the extracts were measured by Folin–Ciocalteu colorimetric method with a minor modification (Slinkard & Singleton, 1977). The detailed process was performed according to Cheng et al. (2020). The absorbance of each sample was measured at 765 nm using a spectrophotometer (UV‐1750; Shimadzu). Phenolic content was expressed as mg gallic acid equivalent (GAE) in dried samples.

### 2.8 HPLC analysis of phenolic compounds

Phenolic compounds of FPs, EPs, GPs, and IBPs were analyzed according to the method from Cheng et al. (2020). The equipment is as follows: an Agilent 1200 series HPLC (Agilent Technologies Ltd), a Zorbax C18 column (4.6 × 150 mm, particle size 5 μm; Agilent). The mobile phase consisted of acetonitrile (A) and water/formic acid (99.8:0.2, v/v) (B). Gradient elution was performed as follows: 0–6 min, 5%–10% A; 6–12 min, 10%–13% A; 12–16 min, 13%–15% A; 16–24 min, 15%–24% A; 24–30 min, 34%–16% A. The injection volume was 10.0 μl and the flow rate was kept at 1 ml/min. The column was operated at 30°C and the detection wavelength was set at 280 nm. Quantification of phenolic compounds of FPs, EPs, GPs, and IBPs was carried out by an external standard method using calibration curves.

### 2.9 Determination of antioxidant activity

#### 2.9.1 DPPH assay

DPPH^−^ scavenging activity of each fraction was evaluated according to the method from Amarowicz et al. (2008). Extracts with 200 μl were mixed with 3.0 ml DPPH solution (200 μmol/L) and placed in the dark for 60 min at room temperature. The absorbance was measured at 517 nm by a spectrophotometer (UV‐1750, Shimadzu).

#### 2.9.2 FRAP and ABTS assay

The values of FRAP and ABTS were measured according to the instruction of the FRAP and ABTS kits.

### 2.10 Statistical analysis

All experiments were repeated three times, and the results are presented as mean ± standard deviation (*SD*). Data were analyzed by SPSS software 19.0 and *p* < .05 was considered a significant difference.

## 3 RESULTS AND DISCUSSION

### 3.1 Observation of appearance and microstructure

**FIGURE 2:**
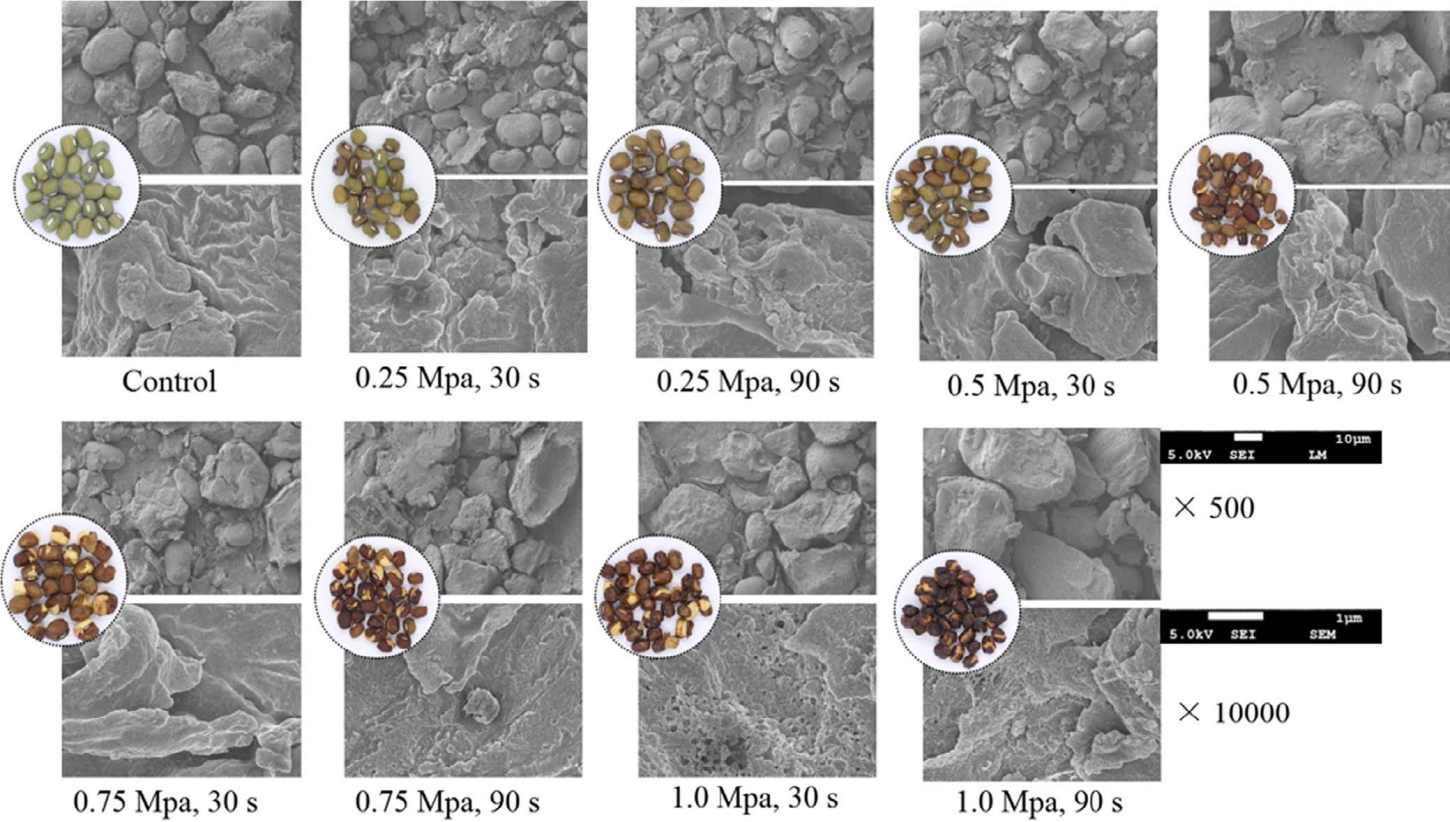
Scanning electron micrographs of mung beans with different steam explosion conditions

After SE, the appearance and microstructure of mung bean were observed by scanning electron microscope (SEM) as shown in Figure 2. Seen from the appearance of mung beans, the increase in the explosion pressure and residence time gradually darkened the color of the mung bean coat. A combination of high temperature and pressure produced by SE can cause Maillard reaction between hydroxyl‐compounds and amino‐compounds, which makes the mung bean coat darker (Sui et al., 2019). The drastic explosion condition can cause the phenomenon of blasting and cracking in mung beans and generate some minuscule cavities and fissures in the bean matrix to make the beans loose and porous. In addition, steam with a powerful seepage force penetrates the interior cells of mung beans, and the huge shear force from an explosive decompression (within 0.0875 s) will make the beans to crack. In contrast, the surface of the untreated beans is relatively regular, compact, and smooth, and no cavities and fissures appear. An increasing porosity is beneficial for the penetration of solvent and to maximize the solubility of active components during the extraction processing (Chen & Chen, 2011; Wang et al., 2021). Similarly, micropores and smaller particles due to SE treatment were conducive for flavonoid extraction by increasing the specific surface area of the material (Song et al., 2014).

### 3.2 Determination of total phenolic contents of four fractions

The contents of FPs, EPs, GPs, and IBPs in mung beans were determined by Folin–Ciocalteu colorimetric method. As shown in Table 1, among the four fractions, IBPs were the dominating fraction, followed by GPs and FPs, and the lowest was EPs. In the untreated group, the contributions of FPs, EPs, GPs, and IBPs to total phenolics were 25.2%, 9.6%, 26.2%, and 39.0%, respectively. The high contribution of the insoluble‐bound form to the total phenolic content was also evaluated in grape byproducts (de Camargo et al., 2014), blackberry (Ayoub et al., 2016), kidney beans (Wang et al., 2016), and spring bay beans (Wang et al., 2016). The contents of free phenolics, soluble conjugate phenolics, and insoluble‐bound phenolics in mung beans were 0.369, 0.201, and 0.250 mg GAE/mg, respectively, and the total phenolic content was only 0.821 mg GAE/mg (Wang et al., 2016), the above contents being well below our results. The content difference may be attributed to experimental materials and extraction methods. The insoluble‐bound form was also the highest content in different tissues (pulp, seed, and peel) of jujube, but the content of the free form was the lowest (Wang et al., 2011). The compositions and distribution of phenolic acids (including free, ester, glycoside, and ester‐binding forms) were also compared in Ponkan and Huyou varieties (Xu et al., 2008).

**TABLE 1:** Total phenolic contents in mung beans with different steam explosion conditions

Treatment	Phenolic acid fraction (mg gallic acid equivalent/g DW)				
	Free phenolics	Esterified phenolics	Glycosided phenolic acids	Insoluble‐bound phenolics	Total phenolics
Control	1.21 ± 0.06^b^	0.46 ± 0.07^a^	1.26 ± 0.02^a^	1.87 ± 0.12^a^	4.80^a^
0.25 MPa, 30 s	1.24 ± 0.07^b^	0.57 ± 0.04^b^	1.34 ± 0.03^a^	2.16 ± 0.06^b^	5.31^b^
0.25 MPa, 90 s	1.30 ± 0.0^bc^	0.70 ± 0.03^c^	1.51 ± 0.03^b^	2.34 ± 0.05^c^	5.85^c^
0.5 MPa, 30 s	1.36 ± 0.06^c^	0.75 ± 0.03^c^	1.54 ± 0.05^b^	2.59 ± 0.06^d^	6.24^d^
0.5 MPa, 90 s	1.79 ± 0.07^f^	0.82 ± 0.04^d^	2.08 ± 0.06^f^	2.61 ± 0.06^d^	7.30^f^
0.75 MPa, 30 s	1.77 ± 0.02^f^	0.86 ± 0.05^d^	2.18 ± 0.02^e^	2.76 ± 0.06^e^	7.57^g^
0.75 MPa, 90 s	1.64 ± 0.09^e^	0.76 ± 0.06^c^	1.91 ± 0.03^d^	2.39 ± 0.05^c^	6.70^e^
1.0 MPa, 30 s	1.50 ± 0.06^d^	0.61 ± 0.03^b^	1.79 ± 0.05^cc^	2.28 ± 0.05^bc^	6.18^d^
1.0 MPa, 90 s	1.09 ± 0.05^a^	0.55 ± 0.02^b^	1.51 ± 0.04^b^	1.76 ± 0.14^a^	4.91^a^

The values in the same row with unlike letters indicate significant differences (*p* < .05)

Steam explosion has a significant effect on the phenolic contents of four fractions in mung beans. The contributions of FPs, EPs, GPs, and IBPs in mung beans with the different SE conditions were 21.8%–25.2%, 9.6%–13.0%, 24.7%–30.7%, and 34.5%–44.1%, respectively. Explosion pressure significantly enhanced the yield of four fractions under the pressure range of 0.25–0.5 MPa. However, a downward trend was found when the pressure increased to 0.75 MPa. Likewise, residence time significantly affected the yield of four fractions. At the range of 0.25–0.5 MPa, the yield with the treatment of 90 s was higher than that of 30 s, but it was the opposite at 0.75–1.0 MPa. In all, the highest yield of phenolics was found at 0.75 MPa for 30 s. Total phenolic content is the sum of the four fractions of phenolics. SE increased the yield of total phenolics in the mung beans, and the highest content (7.57 mg GAE/g DW) was detected at 0.75 MPa for 30 s. Previous reports showed that phenolic contents with acetone‐water extracts ranged from 1.86 to 5.07 mg GAE/g in 10 commercial mung beans, while the phenolic contents in methanolic extracts ranged from 0.08 to 0.28 mg GAE/g (Zhang et al., 2013). On the contrary, phenolic contents in methanolic extracts reached 6.2–10.4 mg GAE/g dry weight in four cultivars of mung beans (Anwar et al., 2007).

Steam explosion can alter the compact texture of biomass via the mechanical and chemical effects (Martin‐Sampedro et al., 2014). In view of molecular structure, SE can cause the hydrolysis of glycosidic bonds and the cleavage of β‐O‐4 ether linkage in lignin, showing the effect of acid‐based hydrolysis (Gong et al., 2012). Therefore, SE can be used as an effective pretreatment technology by directly breaking the ester/ether bond of phenolics and polysaccharides or by hydrolyzing the cell wall matrix to release insoluble bound phenolic acids from cell vacuoles (Chen & Chen, 2011; Gong et al., 2012). In addition, SE is an autohydrolytic process, and a certain amount of organic acids can be formed from cellulose matrix under the combined effects of saturated steam and high temperature (Bonfiglio et al., 2021; Nie et al., 2021). Based on the diversity of plant biomass, the optimal condition of SE is determined by the physical characteristics of samples, and also depends on the pretreatment strategy. The optimal condition of SE on FPs and soluble‐conjugate phenolic acids in barley bran was at 220°C for 120 s (Gong et al., 2012). The highest phenolic compound content in soybean seed coats was obtained at the SE condition of 1.5 MPa for 150 s (Chen et al., 2020), and SE maximally increased the tea polyphenols yield by 15.5% compared with the untreated group (Sui et al., 2019). At the SE condition of 0.8 MPa for 5 min, total flavonoids and phenolic contents in wheat bran increased by 198%, 83%, respectively (Feng et al., 2020). Many studies have also confirmed that SE has a similar effect on phenolic yield and antioxidant activity in different biomass (Cheng et al., 2020; Gong et al., 2021; Hong et al., 2020; Kurosumi et al., 2007). In general, the effect of SE on the phenolics extraction showed a trend of increasing first and decreasing later, and finally, it can reach a balanced state in dissolution and degradation of active compounds (Chen & Chen, 2011; Noda et al., 2013). Excessive explosion treatment (e.g., high temperature/pressure or longer residence time) may result in hydrolysis or polymerization of phenolic compounds to some extent or even carbonization of plant biomass (Stadler et al., 1996).

### 3.3 Identification of phenolics with four forms in mung beans

The individual compounds of FPs, EPs, GPs, and IBPs in mung beans were identified by HPLC. As shown in Table 2, five phenolic compounds including catechin, caffeic acid, epicatechin, *p*‐coumaric acid, and ferulic acid were detected in FPs. Catechin and epicatechin were the dominating compounds in phenolics, followed by caffeic, and *p*‐coumaric and ferulic acids contents were <10 μg/g in the control. After SE, a tendency of first increasing and then decreasing was detected in the yields of phenolic compounds including catechin, epicatechin, *p*‐coumaric acid, and ferulic acid. Conversely, it was an obvious reduction in the content of caffeic acid.

**TABLE 2:** Identification of phenolic compounds in mung beans with the different steam explosion conditions (μg/g)

Content of phenolic compounds	Control	0.25 MPa 30 s	0.25 MPa 90 s	0.5 MPa 30 s	0.5 MPa 90 s	0.75 MPa 30 s	0.75 MPa 90 s	1.0 MPa 30 s	1.0 MPa 90 s
FPs									
Gallic	nd	nd	nd	nd	nd	nd	nd	nd	nd
Protocatechuic	nd	nd	nd	nd	nd	nd	nd	nd	nd
Catechin	25.1 ± 2.8^a^	56.06 ± 13.97^c^	63.36 ± 1.17^d^	56.45 ± 1.31^c^	55.42 ± 0.31^c^	57.69 ± 0.56^c^	47.08 ± 0.37^b^	40.33 ± 1.41^b^	30.8 ± 0.83^a^
Caffeic	12.12 ± 0.45^e^	8.74 ± 0.82^d^	8.18 ± 0.79^c^	8.64 ± 0.38 cd	6.41 ± 0.4^b^	6.64 ± 0.64^b^	5.09 ± 0.49^a^	4.74 ± 0.47^a^	nd
Epicatechin	29.34 ± 2.97^a^	53.95 ± 4.22^e^	52.88 ± 3.17^e^	48.19 ± 1.44^d^	40.06 ± 0.43^c^	41.69 ± 1.3^c^	34.5 ± 0.61^b^	37.02 ± 0.19^b^	29.65 ± 1.63^a^
*p*‐coumaric	9.17 ± 0.38^b^	10.85 ± 0.31^c^	11.14 ± 0.17^c^	12.41 ± 0.17^d^	11.35 ± 0.19^c^	11.17 ± 0.4^c^	9.77 ± 0.11^b^	9.39 ± 0.17^b^	6.30 ± 0.21^a^
Ferulic	5.69 ± 0.84^a^	7.45 ± 0.26^c^	8.28 ± 0.29^d^	7.96 ± 0.09 cd	7.16 ± 0.09^c^	7.35 ± 0.18^c^	6.19 ± 0.18^b^	6.22 ± 0.13^b^	5.52 ± 0.14^a^
EPs									
Gallic	nd	nd	nd	nd	nd	nd	nd	nd	nd
Protocatechuic	nd	nd	nd	nd	nd	nd	nd	nd	nd
Catechin	nd	nd	nd	nd	nd	nd	nd	nd	nd
Caffeic	9.01 ± 0.51^f^	8.27 ± 0.14^e^	7.27 ± 0.17^d^	7.06 ± 0.14 cd	6.56 ± 0.15^bc^	6.41 ± 0.17^b^	6.00 ± 0.08^b^	5.48 ± 0.17^ab^	4.98 ± 0.18^a^
Epicatechin	nd	nd	nd	nd	nd	nd	nd	nd	nd
*p*‐coumaric	18.59 ± 0.21^b^	19.43 ± 0.23^bc^	20.46 ± 0.16^c^	20.78 ± 0.18^c^	21.07 ± 0.32^c^	20.05 ± 0.28^c^	13.22 ± 0.32^a^	18.89 ± 0.12^b^	17.2 ± 0.14^b^
Ferulic	9.20 ± 1.37^a^	10.05 ± 0.48^b^	11.7 ± 0.16^b^	13.34 ± 0.51^c^	15.48 ± 0.58^d^	15.03 ± 0.77^d^	19.37 ± 0.28^e^	12.37 ± 0.4^c^	8.72 ± 0.59^a^
GPs									
Gallic	nd	nd	nd	nd	nd	nd	nd	nd	nd
Protocatechuic	159.13 ± 3.99^a^	160.21 ± 2.75^a^	187.59 ± 0.54^b^	182.1 ± 2.12^b^	194.67 ± 1.55^c^	202.46 ± 1.54^d^	174.67 ± 3.08^b^	182.36 ± 1.25^b^	154.67 ± 6.6^a^
Catechin	nd	nd	nd	nd	nd	nd	nd	nd	nd
Caffeic	nd	nd	nd	nd	nd	nd	nd	nd	nd
Epicatechin	nd	nd	nd	nd	nd	nd	nd	nd	nd
*p*‐coumaric	65.12 ± 3.73^a^	68.43 ± 0.87^a^	78.58 ± 0.57^d^	76.09 ± 0.71^c^	76.07 ± 0.81^c^	79.6 ± 0.95^d^	73.43 ± 0.55^b^	71.61 ± 0.81^b^	66.26 ± 0.39^a^
Ferulic	nd	nd	nd	nd	nd	nd	nd	nd	nd
IBPs									
Gallic	12.71 ± 0.06^b^	14.34 ± 0.38c	13.12 ± 0.34^b^	13.65 ± 0.13^bc^	13.48 ± 0.08^bc^	12.98 ± 0.15^b^	11.91 ± 0.23^b^	12.11 ± 0.11^b^	10.72 ± 0.21^a^
Protocatechuic	148.21 ± 6.41^a^	149.85 ± 7.39^a^	189.44 ± 4.11^c^	178.92 ± 4.39^b^	188.31 ± 1.71^c^	212.31 ± 2.18^d^	185.44 ± 1.49^c^	186.15 ± 0.55^c^	156.00 ± 3.88^a^
Catechin	19.18 ± 0.24^a^	20.62 ± 1.2^a^	24.89 ± 1.07^b^	27.81 ± 0.49^c^	26.68 ± 0.31^c^	26.48 ± 0.16^c^	24.33 ± 0.21^b^	24.52 ± 0.48^b^	18.66 ± 0.65^a^
Caffeic	18.47 ± 0.45^e^	18.04 ± 0.5^e^	16.82 ± 0.17^d^	16.37 ± 0.14^d^	15.98 ± 0.08^c^	14.88 ± 0.09^c^	13.68 ± 0.14^b^	13.14 ± 0.13^b^	10.80 ± 0.13^a^
Epicatechin	33.17 ± 1.26^a^	39.53 ± 1.07^b^	43.37 ± 0.8^c^	50.62 ± 0.84^d^	48.54 ± 0.33^d^	47.92 ± 0.36^d^	43.14 ± 0.49^c^	43.94 ± 0.26^c^	38.48 ± 0.49^b^
*p*‐coumaric	27.84 ± 0.63^a^	28.89 ± 0.15^a^	30.05 ± 0.11^b^	30.52 ± 0.13^b^	31.2 ± 0.32^b^	29.3 ± 0.19^ab^	28.24 ± 0.06^a^	28.11 ± 0.05^a^	27.53 ± 0.22^a^
Ferulic	nd	nd	nd	nd	nd	nd	nd	nd	nd

Abbreviation: nd, no detected.

The values in the same row with unlike letters indicate significant differences (*p* < .05)

For EPs, only three phenolic acids including caffeic, *p*‐coumaric, and ferulic acid were detected; among them, *p*‐coumaric acid was the dominating compound. With the increased severity of SE, the content of *p*‐coumaric and ferulic acids increased first and decreased later. But there was a decrease in the content of caffeic acid after SE.

Both protocatechuic and *p*‐coumaric acids were detected in GPs, but the contents of these two phenolics were relatively high, with the contents exceeding 150 μg/g and 50 μg/g, respectively. SE increased the content of protocatechuic and *p*‐coumaric acid, but the yield of two phenolic acids shows a tendency of first increasing and then decreasing with increasing severity.

For IBPs, six phenolics including gallic, protocatechuic, catechin, caffeic, epicatechin, and *p*‐coumaric were detected in IBPs from mung beans. The results showed that protocatechuic content was the highest, followed by epicatechin, *p*‐coumaric, catechin, caffeic, and gallic in a decreasing order. After SE, an increasing trend was identified in contents of gallic, protocatechuic, epicatechin, and *p*‐coumaric acid; conversely, there was an obvious decrease in caffeic acid.

By comparison, gallic acid only exists in the insoluble‐bound form, protocatechuic is in glycosidic and insoluble‐bound forms, catechin is in esterified and insoluble‐bound forms, ferulic is in both free and esterified forms, caffeic does not exist in glycosidic form, and *p*‐coumaric is in all four forms. The phenolic compounds of free, soluble conjugate, and insoluble‐bound phenolics in mung beans were also identified and quantified (Wang et al., 2016), and the results showed that the free form mainly includes epicatechin, *p*‐coumaric, ferulic, rutin, isoquercitrin; the conjugate form includes gallic acid, protocatechuic, caffeic, syringic, *p*‐coumaric, ferulic, rutin, isoquercitrin, quercitrin; insoluble‐bound form includes gallic acid, protocatechuic, catechin, caffeic, syringic, *p*‐coumaric, ferulic, rutin, isoquercitrin. The difference in phenolic composition and content is mainly due to different extraction methods and materials. Studies also confirmed that mung beans were rich in flavonoids, mainly including vitexin and isovitexin, with the highest contents of 1.50 and 1.10 mg/g, respectively (Zhang et al., 2013).

Steam explosion has a significant effect on the compositions and contents of different combined phenolic acids (Chen, Zhang, et al., 2016; Gong et al., 2012; Sui et al., 2019). After the SE treatment of wheat bran, the release of free and bound ferulic acids has been effectively increased (Chen, Chen, et al., 2016). Similarly, some bound polyphenols were easier to be released from cereal cell walls after SE; *p*‐coumaric acid and ferulic acid in their free and conjugated forms are more easily destroyed by SE (Gong et al., 2012). The increase in soluble ferulic acids may be partly attributed to the breaking of glycosidic or ester/ether bonds of biomass caused by SE (Gong et al., 2012, 2016). In addition, ferulic acid and *p*‐coumaric acid can be decarboxylated to form dimerized products under thermal conditions, and ferulic acid may be degraded into other substances (e.g., 4‐methyl‐, 4‐ethyl‐ and 4‐vinylguaiacol, vanillin) after excessive steam treatment (Fiddler et al., 1967). These phenomena indicated the combination of chemical effect and mechanical force resulting from SE occurred to a balance of formation and decomposition in phenolic compounds.

**FIGURE 3:**
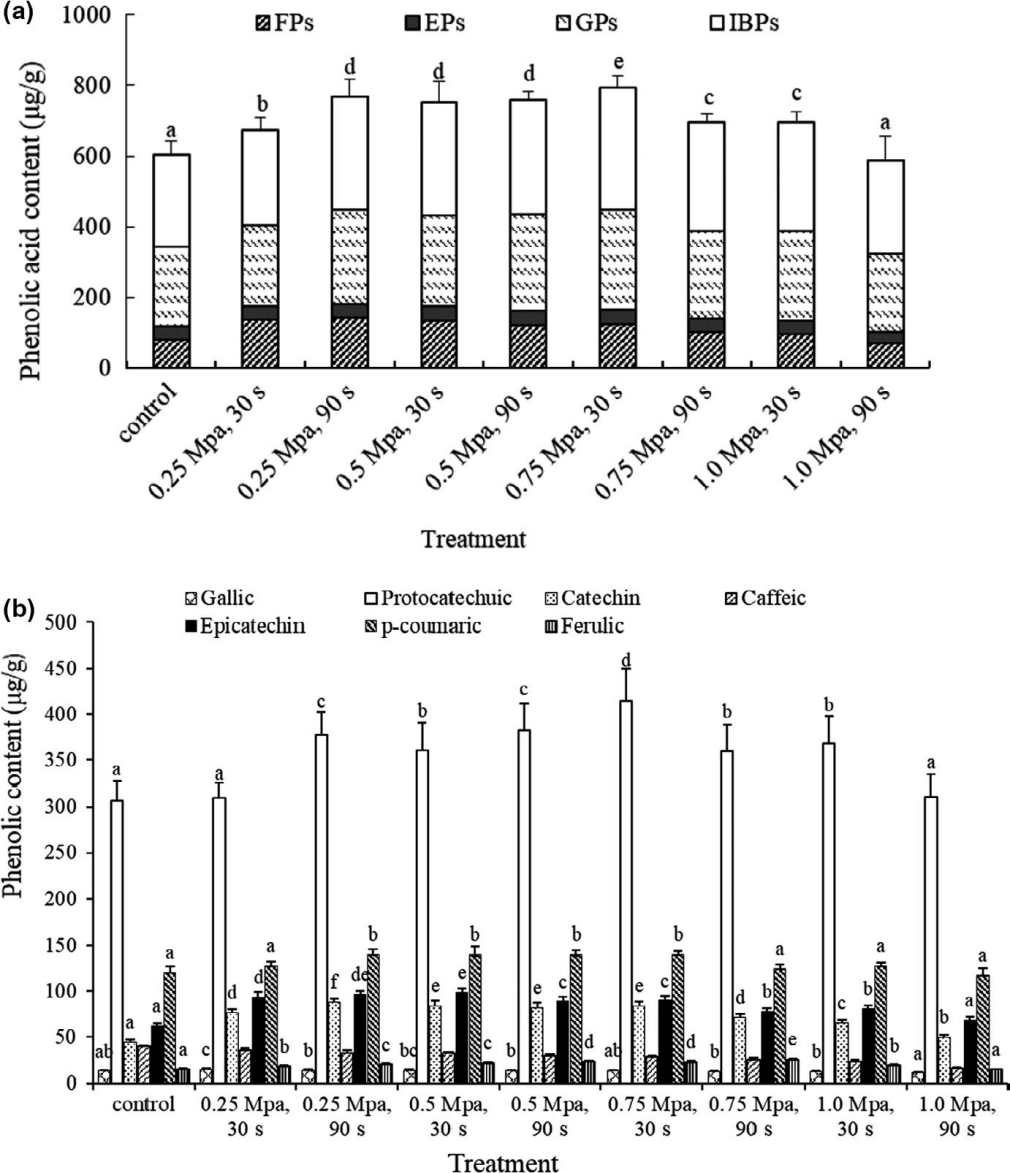
Distribution of phenolics in mung beans with the different steam explosion conditions. (a) Total contents of individual phenolic; (b) Phenolic contents in four forms. Different capital letters above column indicate significant differences between groups (*p* < .05)

According to the data in Table 2, the sum of individual phenolics in the four forms is referred to as the total content of each phenolic, as shown in Figure 3a. Protocatechuic was the main phenolic component of mung beans, followed by *p*‐coumaric acid and epicatechin; the lowest content was gallic acid and ferulic acid. The highest content of protocatechuic acid and ferulic acid was detected at 0.75 MPa for 30 s.

According to the data in Table 2, the sum of all phenolics in each form is referred to as the total content of this form as shown in Figure 3b. The proportion of the esterified form is the lowest in the four forms and followed by the free form, which is similar to the result from Table 1. With the increasing strength of SE, a tendency of first increasing and then decreasing was displayed in the total content of phenolics from the four forms, and the highest total content was observed at 0.75 MPa for 30 s.

### 3.4 Effect of SE on the antioxidant activity

**FIGURE 4:**
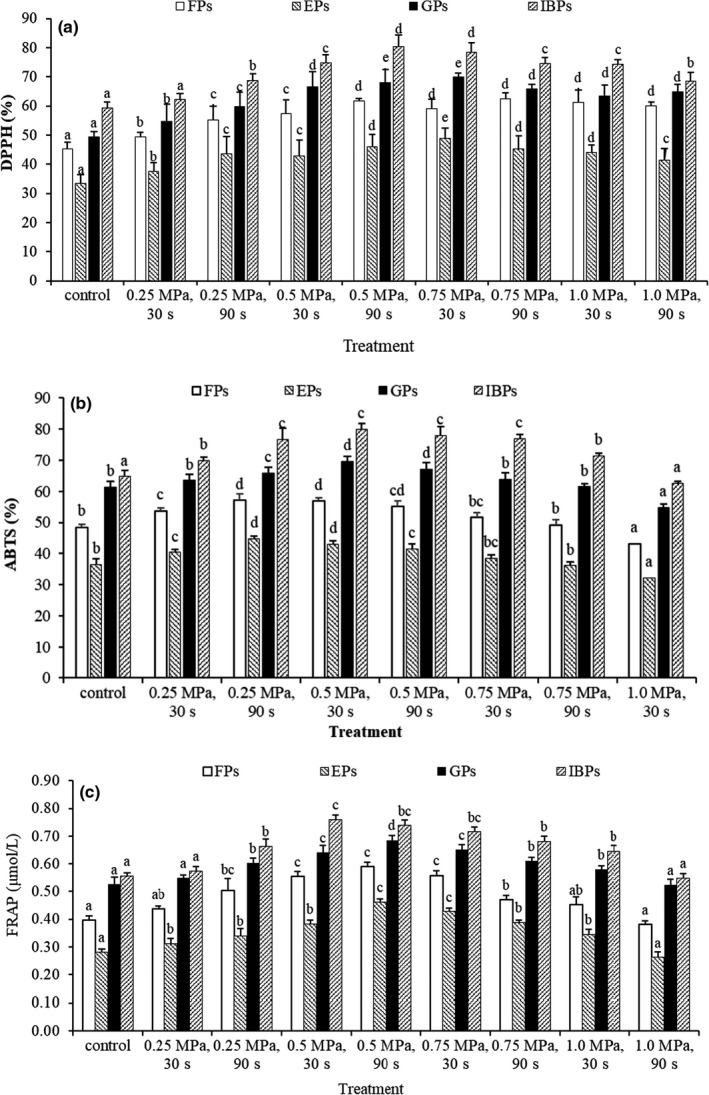
Antioxidant activity of each phenolic fraction in mung beans with the different steam explosion conditions. (a, 2,2′‐diphenyl‐1‐picrylhydrazyl [DPPH]; b, 2,2′‐azinobis (3‐ethylbenzothiazoline‐6‐ sulfonic acid) [ABTS]; c, ferric reducing antioxidant power [FRAP]). Different capital letters above column indicate significant differences between groups (*p* < .05)

The antioxidant activity of FPs, EPs, GPs, and IBPs in mung beans with different SE conditions was measured by using the DPPH, ABTS, and FRAP assays, respectively, as shown in Figure 4. Obviously, SE improved the antioxidant activity of the four forms in mung beans. Residence time also enhanced the antioxidant activity of four fractions in the range 0.25–0.5 MPa. On the contrary, the long residence time of 90 s decreased the antioxidant activity at 0.75–1.0 MPa. DPPH, ABTS, and FRAP assays in different treatments appeared to have similar trends, with high antioxidant activity in IBPs and low antioxidant activity in EPs. A higher antioxidant activity of insoluble‐bound phenolics from mung beans was also exhibited than those of free and soluble conjugates phenolics by FRAP, DPPH, and TEAC (trolox equivalent antioxidant capacity) assays (Wang et al., 2016). On the contrary, free and insoluble‐bound phenolics from swallow root showed a lower FRAP radical scavenging capacity compared with soluble conjugates (Harish Nayaka et al., 2010). Similarly, DPPH radical scavenging capacity of insoluble‐bound phenolics from edible flowers was lower than their soluble counterparts (Kaisoon et al., 2011). The antioxidant activity of phenolics from four mung bean cultivars was compared by using reducing power and bleaching β‐carotene in linoleic acid system (Anwar et al., 2007). It can be said that mung bean extracts showing a stronger antioxidant activity is partly attributed to the rich phenolics in extracts (Anwar et al., 2007; Wang et al., 2016; Zhang et al., 2013). SE increased the polyphenol content in mung bean extracts, and correspondingly, their antioxidant activity was significantly improved. The content of protocatechuic, caffeic, epicatechin, and gallic acids is obviously related to the free radical scavenging ability by FRAP, DPPH, and ABTS assays, and the total phenol content also has a significant correlation with FRAP and DPPH (Chen, Chen, et al., 2016; Cheng et al., 2020). The FRAP and •OH,${}^{\cdot }O_{2}^{‐}$ radical scavenging capacity proved at least 20% enhancement of the antioxidant capacity of tea wastes after SE (Sui et al., 2019). Similarly, explosion pressure and residence time also improved the antioxidant activity of phenolic from the soybean seed coats (Chen et al., 2020). These results had demonstrated that there is a dose‐dependent relationship between the “total phenolic content” and “antioxidant activity.”

The relationship of phenolics in four forms of mung beans and their antioxidant activities was investigated, and the correlation coefficients (*R* ^2^) are shown in Table 3. As can be seen, the correlation coefficients of phenolic content and their antioxidant activities were ABTS > DPPH > FRAP, and correlation coefficients in each assay were very high (*R* ^2^ = 0.911, 0.893, and 0.877 for ABTS, DPPH, and FRAP, respectively). Moreover, there was a high positive correlation between DPPH and ABTS, between DPPH and FRAP, and between ABTS and FRAP, that is, the correlation coefficients (*R* ^2^) of 0.851, 0.832, and 0.954, respectively. Likewise, the total phenol content in soybean seed coats is highly correlated with their antioxidant activity including DPPH and ABTS (Sui et al., 2019). SE significantly enhanced the antioxidant activity, and the relationship between phenolic content and antioxidant activity was very high (Feng et al., 2021; Hu et al., 2020). These results revealed that enhancement of antioxidant activity may be attributed partly to the increase in the concentration of phenolic compounds. On the contrary, the relationship is not obvious between total phenolic content and reducing power (*r* = 0.478), total phenolic content, and bleaching of β‐carotene assay (*r* = 0.381) (Anwar et al., 2007). These results from different antioxidant assays showed different degrees of correlation, indicating that not a single method is adequate to evaluate the antioxidant activity of plant extracts.

**TABLE 3:** Correlation analysis of phenolics and antioxidant activity

	DPPH	ABTS	Ferric reducing antioxidant power
Phenolic content	0.893	0.911	0.877
DPPH		0.851	0.832
ABTS			0.954

Abbreviations: ACTS, 2,2′‐azinobis (3‐ethylbenzothiazoline‐6‐ sulfonic acid); DPPH, 2,2′‐diphenyl‐1‐picrylhydrazyl.

## 4 CONCLUSION

Compared with the untreated sample, SE pretreatment was used to alter the pore structure of mung beans, which eventually increased the yield of active ingredients and enhanced their functionality. IBPs were the dominating fraction in mung beans, and GPs, FPs, and EPs were in a decreasing order. Protocatechuic was the most affluent phenolic acid, whereas ferulic and gallic acid were the least in mung beans. After SE, a significant increase appeared in the yield of FPs, GPs, EPs, and IBPs. Individual compounds identified by HPLC showed that an increasing tendency was investigated in contents of gallic, protocatechuic, epicatechin, and *p*‐coumaric acid, but the content of caffeic acid clearly declined. Thereby, SE also heightened the antioxidant activity of four forms. The phenolic content was positively correlated with antioxidant activity (ABTS, DPPH, FRAP). With increasing SE intensity, a trend of first increasing and then decreasing was investigated in the yield of phenolics and their antioxidant activity. In all, the optimal explosion condition for the extraction of phenolics and the strong antioxidant activity in mung beans were at a pressure of 0.75 MPa for 30 s. Hence, it can be concluded that SE under the optimal condition can maximally enhance the release of phenolic compounds and improve their antioxidant activity in mung beans.

## CONFLICT OF INTEREST

The authors have declared no conflict of interest.

## Data Availability

The data used to support the findings of this study are included in the article and are available from the corresponding author upon reasonable request.
